# Tobacco market trends in 97 countries between 2007 and 2021

**DOI:** 10.18332/tid/177441

**Published:** 2024-02-02

**Authors:** Yifei Liu, Filippos T. Filippidis

**Affiliations:** 1 Department of Primary Care and Public Health, School of Public Health, Imperial College London, United Kingdom

**Keywords:** tobacco control, retail value, market share, market trends

## Abstract

**INTRODUCTION:**

Analysis of the tobacco market can provide valuable insights for developing tobacco control strategies. This study examines the market trends of cigarettes, cigars/cigarillos, smoking tobacco, smokeless tobacco, e-cigarettes, heated tobacco products (HTPs), and tobacco-free oral nicotine, across 97 countries between 2007 and 2021.

**METHODS:**

We obtained annual tobacco retail value data from Euromonitor Passport and calculated the market share for each type of tobacco product. The research examined trends in retail value and market share globally, stratified by national income level, as well as in individual countries.

**RESULTS:**

From 2007 to 2015, the growth of the global tobacco market was primarily driven by cigarette sales. However, starting in 2016, emerging products, including e-cigarettes, HTPs, and tobacco-free oral nicotine, as well as non-cigarette combustible products, including cigars/cigarillos and smoking tobacco, have been mostly responsible for the increases in the global tobacco retail value. High-income countries experienced the greatest increase in the retail value of emerging products, while middle- and low-income countries still observed rises in cigarette sales.

**CONCLUSIONS:**

Trends in the retail value of different tobacco products varied widely during the study period, with distinct trends observed in different income levels and within individual countries. These trends can supplement prevalence data and be used to inform local tobacco control policies.

## INTRODUCTION

Between 2000 and 2020, the global prevalence of tobacco smoking decreased from 27% to 17%^1^. However, the tobacco and nicotine market has been disrupted by the introduction of emerging tobacco or nicotine products, such as e-cigarettes and heated tobacco products (HTPs)^2^. These are often promoted by the tobacco industry (TI) as ‘reduced-risk products’, which can be particularly appealing to the growing number of smokers who are now aware of the health risks associated with tobacco use, even though the risk of these products is not zero, and they are addictive^3,4^. Moreover, the flavors and stylish designs of many emerging tobacco or nicotine products make them very attractive to young people^5,6^. As a result, concerns arise that these emerging products could act as a gateway to initiating smoking of conventional tobacco products but also renormalize nicotine use in the younger generations, even in countries where smoking in youth has declined substantially over the past decades^7^.

Nevertheless, in 2020, 91% of all tobacco smokers globally were still using cigarettes^1^. Apart from cigarettes, the use of other traditional types of tobacco products remains a serious public health issue. For instance, the global prevalence of chewing tobacco use increased slightly from 1990 to 2019, with >83% of users (228 million) living in South Asia, where smokeless tobacco has always been very popular^8^. The use of waterpipe tobacco has also grown in popularity recently, particularly in Europe and the Eastern Mediterranean^9^. Moreover, many smokers in countries with high cigarette taxes are switching to cheaper products, such as roll-your-own (RYO) tobacco, to reduce the cost of smoking^10^.

As recommended by the M measure (‘Monitor tobacco use and prevention policies’) in the WHO MPOWER package, it is important to monitor all types of tobacco use in order to track changes in consumer preferences and spot gaps in current regulations^11^. Compared with prevalence data, retail sales data for tobacco products can be collected more easily and can, therefore, provide timely insights into the trends in tobacco use across many countries. Furthermore, tobacco companies prioritize their revenue over the actual number of tobacco users. Therefore, analyzing sales data can be a valuable approach to comprehend the core products and key markets of the TI. This, in turn, enables us to gain insights into the strategies and priorities of tobacco companies.

Within this context, this study aims to explore the tobacco market trends in terms of market size, retail value and market share across 97 countries from 2007 to 2021. It provides an overview and comparison of trends at different levels, including global trends, trends by national income levels, and trends for each country for seven major types of products.

## METHODS

### Data sources

*Tobacco market data*

Data on tobacco retail value from 2007 to 2021 for 97 countries were obtained from Euromonitor International Passport^12^. Euromonitor International categorized tobacco/nicotine products into seven categories, including cigarettes, cigars/cigarillos, smoking tobacco (including pipe tobacco and RYO tobacco), smokeless tobacco, e-cigarettes, HTPs, and tobacco-free oral nicotine. Euromonitor does not include sales data for nicotine replacement therapy (NRT) in the tobacco product market data. Thus, NRT is not included in our analysis. For the purpose of this study, cigarettes, cigars/cigarillos, smoking tobacco, and smokeless tobacco are considered conventional products, while e-cigarettes, HTPs, and tobacco-free oral nicotine are referred to as emerging products. Even though e-cigarettes and oral nicotine are nicotine products that do not contain tobacco, we considered them parts of the broader tobacco market, and we included them in our analysis. The sales data for HTPs and oral nicotine were reported since 2013 and 2018, respectively. The retail value used in this study was measured using constant 2021 prices and fixed 2021 exchange rates from local currency to US dollars to account for inflation and exchange rate fluctuations.

*Country-level sociodemographic data*

We obtained data on the country’s population from the United Nations Population Division^13^. Since the 97 countries included in this study represented 88.4% of the world population in 2021, we refer to the total of these countries as ‘global’. We classified the 97 countries into high-income (n=42), upper middle-income (n=26) and lower middle/low-income (n=29) using the World Bank Country Classification^14^.

### Statistical analysis

The market size of each tobacco product category was described using the retail value data. The market share of a specific product type was defined as the proportion of its retail value in relation to the retail value of all tobacco products. Stacked bar charts were used to illustrate trends in market size and market share from 2007 to 2021, both at the global level and by country income level. Due to the large population and the idiosyncratic characteristics of the tobacco market in China and India, additional line graphs for the retail value of cigarettes and cigars/cigarillos in upper middle-income countries without China and for the retail value of smokeless tobacco in lower middle/ low-income countries without India were plotted. These graphs were plotted in Stata 13.1.

To analyze country-level market share changes, the absolute difference between a product’s market share in 2021 and that in 2007 (or the year when it was introduced in the country) was calculated and presented on maps created in Excel.

Treemaps which show the relative market size (compared to the global market) of a specific product in each country, were plotted in Excel. Treemaps for the four conventional product types for 2007 and 2021 were created. For the emerging product types, 2021 and the years when the product first appeared in at least one-third of the 97 countries were chosen, which were 2011 for e-cigarettes (39 countries) and 2018 for HTPs (33 countries). As for oral nicotine, only the treemap for 2021 is presented (22 countries).

## RESULTS

A total of 5283 retail value data points for the seven categories of tobacco products across the 97 countries were analyzed (Supplementary file Table 1). The 97 countries consist of 42 high-income countries (HICs), 26 upper middle-income countries, and 29 lower middle/low-income countries (Supplementary file Table 1), representing 96.2%, 97.9%, and 80.8% of the total population in their income level, respectively.

### Trends in the global tobacco market

**Figure 1:**
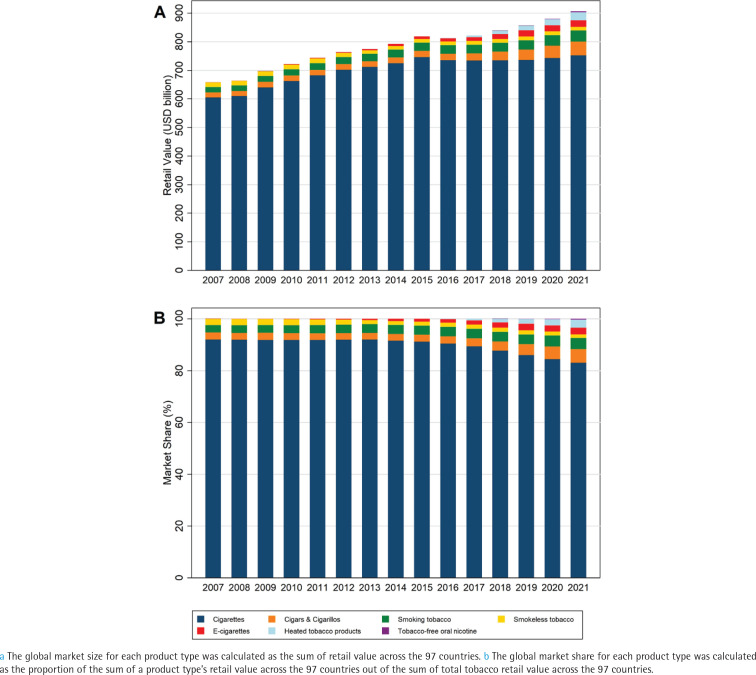
Trends in global market size^a^ and market share^b^ of the seven types of tobacco or nicotine products, for 97 countries, 2007–2021

Between 2007 and 2021, the total value of tobacco sales showed a consistent increase, rising from US$657 billion to US$906 billion across 97 countries. From 2007 to 2015, the growth of the overall market was mainly fueled by cigarette sales. However, since 2016, the growth has been primarily driven by sales of other products, including cigars/cigarillos, smoking tobacco, and the three types of emerging products (Figure 1 A).

Between 2007 and 2015, cigarettes occupied over 91% of the global tobacco market. However, by 2021, their market share had decreased to 83%. On the other hand, the market shares of cigars/cigarillos and smoking tobacco experienced an increase over the 15-year period. E-cigarettes, HTPs, and oral nicotine also witnessed small annual increments in their market shares, although their market shares remained small by the end of the study period, with a share of 2.5%, 3.1%, and 0.3% of the overall tobacco market in 2021, respectively (Figure 1 B).

### Tobacco market trends by income level

**Figure 2:**
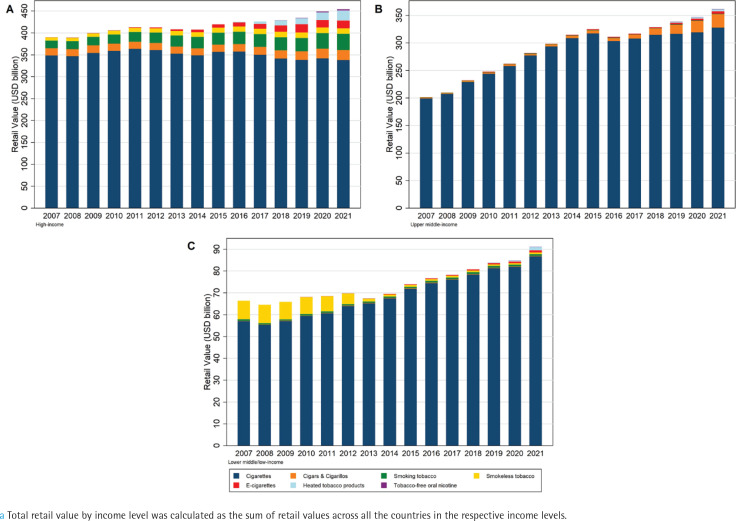
Total retail value^a^ of the seven types of tobacco or nicotine products in: A) high-income countries (N=42); B) upper middle-income countries (N=26); and C) lower middle/low-income countries (N=29), for 97 countries, 2007–2021

The market size for cigarettes in HICs stayed relatively stable over the 15 years, hovering around $350 billion. Meanwhile, the retail value of all other categories experienced consistent growth (Figure 2 A). Thus, we can observe a decline in the market share of cigarettes, from 89.5% to 74.5% (Supplementary file Figure 1 A). It is worth noting that, in comparison to the other two income levels, HICs consistently demonstrated a higher market share of emerging tobacco products. By 2021, the market share for e-cigarettes, HTPs, and oral nicotine in HICs reached 3.9%, 5.1%, and 0.7%, respectively (Supplementary file Figure 1 A).

In upper middle-income countries, the retail value of cigarettes rose from $199 billion to $317 billion from 2007 to 2015, followed by a $14 billion decline in 2016. Subsequently, the growth of cigarette sales slowed down, while the market for cigars/cigarillos grew quickly (Figure 2 B). These trends primarily reflected the patterns observed in China, where the majority of the population in this income-level group resides. In the Chinese market, cigarette sales rose steadily from just over $140 billion in 2007 to more than $260 billion in 2021, with a slight decline in 2016. Moreover, sales of cigars/cigarillos in China increased from about $4.5 billion in 2016 to almost $24 billion in 2021 (Supplementary file Figures 2 A and B). Regarding market share within the upper middle-income group, cigarettes decreased from 98.9% to 90.7% over the 15 years, while cigars/ cigarillos went from 0.6% to 6.8% (Supplementary file Figure 1 B).

Cigarette sales in lower middle/low-income countries experienced a gradual increase from $57 billion to $87 billion from 2007 to 2021. However, the retail value of smokeless tobacco decreased from $8.5 billion in 2007 to less than $1 billion in 2015, with a continued slow decline thereafter (Figure 2 C). This decline was primarily influenced by reduced sales of smokeless tobacco in India (Supplementary file Figure 2 C). As a result, the market share of smokeless tobacco in this income level declined from 12.8% in 2007 to 1.8% in 2013 (Supplementary Figure 1 C).

In both upper middle-income and lower middle/ low-income groups, emerging tobacco products still represented a relatively small proportion of the overall tobacco market (Supplementary file Figures 1 B and C). By 2021, the combined market share of the three categories was 2.2% in upper middle-income countries and 3.1% in lower middle/low-income countries.

### Changes in tobacco market share at the country level

**Figure 3:**
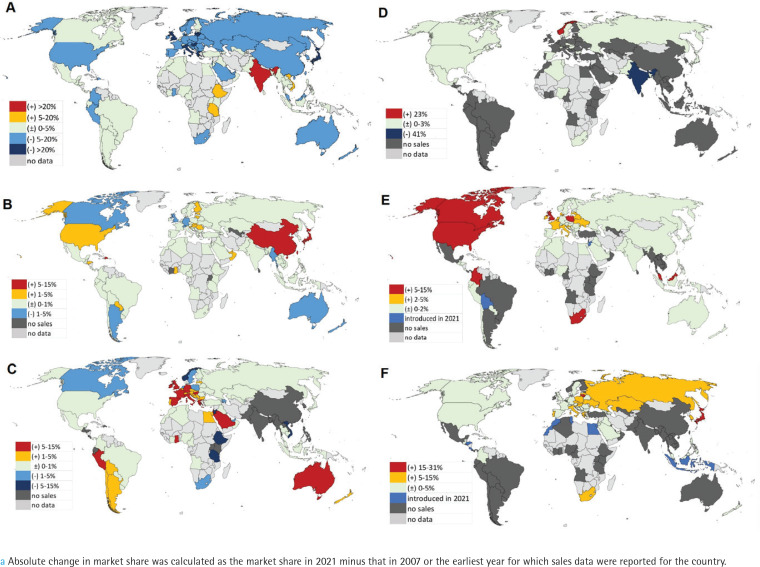
Absolute change in market share^a^ of: A) cigarettes, B) cigars/cigarillos, C) smoking tobacco, D) smokeless tobacco, E) e-cigarettes, and F) heated tobacco products, at the country level across 97 countries, 2007–2021

Over the 15-year period, the market share of cigarettes witnessed a decline in most countries, with Japan and many European countries experiencing the largest decrease. In contrast, India saw the largest increase in market share of cigarettes (+40%) (Figure 3 A). The market share of cigars/cigarillos remained stable in most countries, except for relatively large increases in the Dominican Republic, China, and Japan (Figure 3 B). Smoking tobacco experienced an increase in market share in regions including Western Europe, South America, the Middle East, and Australia, while Norway had the largest decrease (-11%) (Figure 3 C). The market share of smokeless tobacco showed minimal changes overall, but Norway observed the largest increase (+23.4%), and India experienced the largest decrease (-41%) (Figure 3 D). The market share of emerging products increased in most countries where they were available. South Africa recorded the largest increase in e-cigarette market share (+13% from 2008 to 2021), while Japan witnessed the largest increase in HTPs market share (+31% from 2014 to 2021) (Figures 3 E and F). This calculation was not done for oral nicotine because it was only introduced in 2018.

### Relative tobacco market size

**Figure 4:**
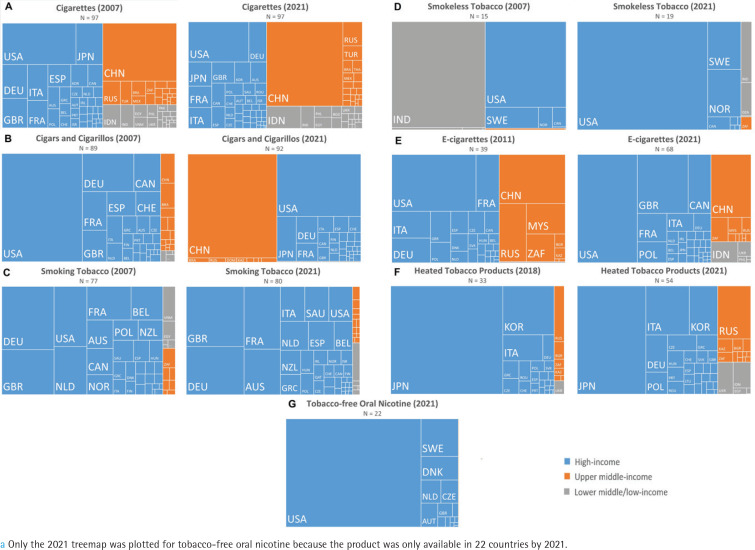
Treemap representation of the relative market size for: A) cigarettes, B) cigars/cigarillos, C) smoking tobacco, D) smokeless tobacco, E) e-cigarettes, F) heated tobacco products, and G) tobacco-free oral nicotine^a^, in each country and each income level group in 2007 (or the year when the product first appeared in at least one-third of the 97 countries) and 2021

For the cigarette market, HICs maintained the largest share of the global market from 2007 to 2021, but China was the largest market at the country level in both years (Figure 4 A). In 2007, the USA held over a third of the global cigars/cigarillos market, and other major markets were mainly European countries. However, China emerged as the largest cigars/ cigarillos market in 2021, followed by the USA and Japan (Figure 4 B). Regarding smoking tobacco, HICs dominated the market throughout the study period, with Germany and the UK holding their positions as the top two markets (Figure 4 C).

Regarding smokeless tobacco, India accounted for more than half of the global market in 2007, but, by 2021, the USA became the largest, followed by Sweden and Norway (Figure 4 D). In addition, oral nicotine was primarily popular in countries with relatively large smokeless tobacco markets, such as the USA and Sweden (Figure 4 G).

HICs were the main markets for e-cigarettes and HTPs. The USA and many European countries held the lead in the e-cigarette market in both 2011 and 2021, while Japan, South Korea, and Italy were the top HTP markets in both 2018 and 2021. In 2021, other major e-cigarette markets included China, South Africa, and Indonesia, while Russia and Ukraine emerged as notable markets for HTPs (Figures 4 E and F).

## DISCUSSION

Globally, the market size of the overall tobacco market, in terms of retail value, continued to expand between 2007 and 2021. However, the market share of cigarettes declined over the period, while other combustible tobacco products and emerging products gained market share. In general, cigarette sales continued to increase in low- and middle-income countries (LMICs), while the retail value of non-cigarette products increased noticeably in HICs.

Over the 15-year period we analyzed, the retail value of cigarettes remained relatively steady in HICs. Given that there was a rise in the real cigarette prices in HICs^15^, it can be inferred that the retail volume has decreased. On the contrary, the cigarette market continued to grow in LMICs over the 15 years. While smoking prevalence had decreased in all income levels between 2000 and 2020, this reduction has not been translated into a reduced number of smokers in many regions because of population growth^1^, which might help explain the rise in cigarette sales in LMICs.

The retail value and market share of cigars/ cigarillos have been rising in upper middle-income countries over the period. This rise was mainly driven by increased sales in China. According to Euromonitor’s country reports, the most popular subtype in China is standard-sized cigars^16^, which are probably the priciest category of tobacco products^17^. Therefore, this growth in China may be attributed to the country’s economic development, leading to higher consumer expenditure on premium tobacco products.

In contrast, HICs clearly dominated the global smoking tobacco market. Previous studies have indicated a rising prevalence of RYO tobacco use in many HICs, such as the USA, UK, France, and Germany^18-20^. RYO tobacco users frequently report that they use it because it is more affordable^20,21^. Thus, the discrepancy between high taxes imposed on cigarettes and relatively lower taxation on RYO tobacco in many HICs may have incentivized price-sensitive cigarette smokers to switch, which led to the observed increase in smoking tobacco’s market share in these settings^19^.

There was a significant decline in both the retail value and market share of smokeless tobacco in India in the early 2010s, while most other countries assessed showed minimal changes during the period. This decline in India was likely due to the ban on gutkha sales implemented in 2011^22^. However, previous research has reported no change in the prevalence of chewing tobacco use in India between 1990 and 2019^8^. This contrasts with our findings, possibly because the Euromonitor data did not capture illegal sales of smokeless tobacco, which has been reported in India before^23^. This emphasizes the importance of considering sales and prevalence data together to obtain a more comprehensive understanding of tobacco use.

As expected, there was a clear upward trend in both retail value and market share in emerging tobacco products, particularly in HICs. There are several reasons why the TI might target HICs for their emerging products. Firstly, in HICs, there is a growing awareness of the health risks associated with tobacco use, making the promotion of these ‘less harmful’ emerging products more appealing to consumers^24,25^. Additionally, certain emerging products, such as HTPs, tend to be priced higher than cigarettes^17^, so they are relatively more affordable in HICs than in LMICs. Interestingly, among HICs, although the USA and European countries dominated the e-cigarette market, the largest markets for HTPs were Japan, South Korea, and Italy. The higher sales of HTPs align with the higher prevalence of HTP use in these countries^24,26-28^ and might be attributed to TI’s pilot initiatives with HTPs in these specific markets^29^.

There has been limited focus on the market for emerging tobacco products in LMICs. However, our findings indicate that, although emerging products are still a small portion of the overall tobacco market in these income levels, the retail value of all three types of emerging products has been increasing. These products are not currently widely available in many LMICs, but it is possible that the TI will begin to expand its business in emerging tobacco products to these countries. LMICs are attractive markets because they often lack strict regulations on emerging products, and they have a substantial number of tobacco users and young people with a rising disposable income^30,31^.

### Strengths and limitations

This multi-country analysis comprehensively examined the trends in both the retail value and market share of various tobacco products from 2007 to 2021. As such, it can serve as an important reference for future research in the field of tobacco control. We could not analyze changes in the sales volume of these products because Euromonitor does not collate these data for all categories of tobacco products. While it is worth noting that not all countries were included, this study covered a substantial portion of the global population. However, the generalizability of the findings to low-income countries could be limited due to the inclusion of only two low-income countries in the analysis. Regarding the methodology used by Euromonitor International to collect the sales data, the specific details were not extensively described, and the company has recently started working with tobacco companies^32^, but the Passport database has been widely utilized in previous tobacco research. Furthermore, the data do not capture illicit trade, which may constitute a significant portion of the tobacco market, as illustrated by the case of smokeless tobacco in India, and can vary across countries^33^. Nevertheless, retail value helps to identify the product types and the countries or regions that generate the most revenue for the TI, which can be valuable information for developing tobacco control strategies. However, caution should be exercised when comparing the market sizes of different products across different countries, as the proportion of revenue going to the TI may differ due to the varying taxes applied to different products in different countries.

## CONCLUSIONS

This study provides a comprehensive overview of the changes in the tobacco market from 2007 to 2021 and the variations in market trends across different income levels and countries. These findings could potentially be used to inform tobacco control policies aimed at curtailing the increasing popularity of specific types of products. For example, although the tobacco industry claims to be transforming to a smoke-free future, it is clear that the increase in sales of smoking tobacco, RYO products, and HTPs, which all contain tobacco, has continued throughout the study period. In response, the European Union recently implemented new regulations on HTPs, including a ban on flavoring and the elimination of the rights of member states to exempt HTPs from displaying health warnings^34^. Continuous monitoring of the tobacco market is important for staying abreast of the emerging trends in tobacco sales and identifying regulatory gaps for effective tobacco control.

## Data Availability

The data supporting this research are available from the authors on reasonable request.
