# Supplementary material for: Tobacco market trends in 97 countries between 2007 and 2021
Source: Tob Induc Dis. 2024 Feb 2;22:10.18332/tid/177441. doi: 10.18332/tid/177441 (PMC10835573; doi:10.18332/tid/177441)
Supplement: Supplementary file 1 [file TID-22-31-s1.pdf]

Supplementary Tables and Figures

Supplementary Table 1. Number of data points and number of countries assessed by product type and by income level, across 97 countries between 2007 and 2021

	High-income[a]		Upper middle-income[b]		Lower middle/low-income[c]		Total	
	Data points	Countries	Data points	Countries	Data points	Countries	Data points	Countries
Cigarettes	630	42	390	26	435	29	1455	97
Cigars/cigarillos	630	42	390	26	338	24	1358	92
Smoking tobacco	615	41	299	21	266	19	1180	81
Smokeless tobacco	136	12	38	4	89	6	263	22
E-cigarettes	438	37	217	21	90	13	745	71
Heated Tobacco Products	152	29	62	17	15	8	229	54
Oral nicotine	40	16	8	4	5	3	53	23

[a] High-income countries (42 countries): Australia, Austria, Belgium, Canada, Chile, Croatia, Czech Republic, Denmark, Estonia, Finland, France, Germany, Greece, Hungary, Ireland, Israel, Italy, Japan, Kuwait, Latvia, Lithuania, Netherlands, New Zealand, Norway, Oman, Panama, Poland, Portugal, Qatar, Romania, Saudi Arabia, Singapore, Slovakia, Slovenia, South Korea, Spain, Sweden, Switzerland, United Arab Emirates, United Kingdom, Uruguay, USA

[b] Upper middle-income countries (26 countries): Azerbaijan, China, Kazakhstan, Malaysia, Thailand, Belarus, Bosnia and Herzegovina, Bulgaria, Georgia, North Macedonia, Russia, Serbia, Argentina, Brazil, Colombia, Costa Rica, Dominican Republic, Ecuador, Guatemala, Mexico, Paraguay, Peru, Iraq, Jordan, South Africa, Turkey

[c] Lower middle- and low-income countries (29 countries): Bangladesh, Cambodia, India, Indonesia, Laos, Myanmar, Pakistan, Philippines, Sri Lanka, Uzbekistan, Vietnam, Ukraine, Bolivia, El Salvador, Honduras, Algeria, Angola, Cameroon, Cote d'Ivoire, Egypt, Ethiopia, Ghana, Kenya, Lebanon, Morocco, Nigeria, Tanzania, Tunisia, Uganda

Supplementary Figure 1. Market share[a] of the seven types of tobacco or nicotine products in (A) high-income countries (N=42), (B) upper middle-income countries (N=26), and (C) lower middle/low-income countries (N=29) from 2007 to 2021

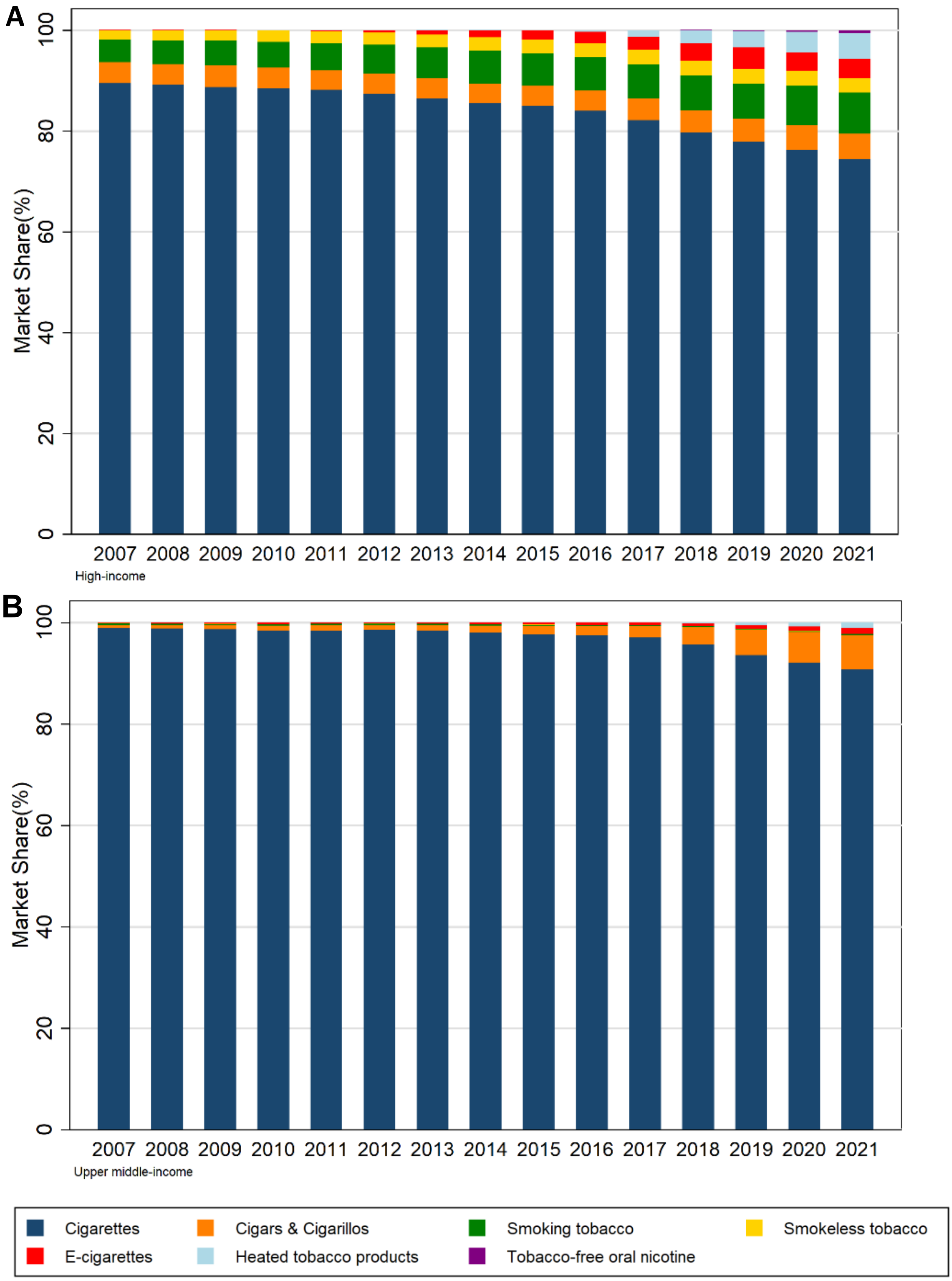

Supplementary Figure 1 continued on next page.

Supplementary Figure 1. (Continued)

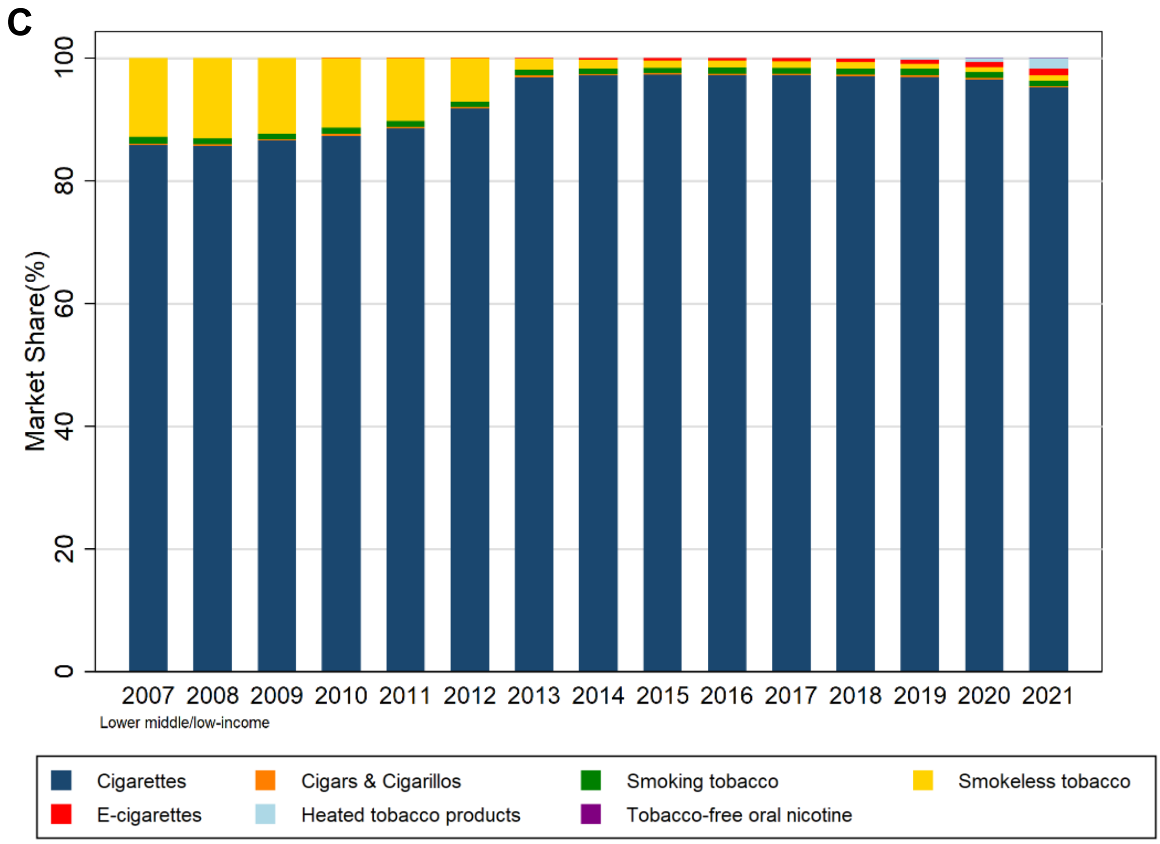

[a] The market share by income level was calculated as the proportion of the sum of a product type's retail value across all the countries in the income level out of the sum of total tobacco retail value across all the countries in the income level.

Supplementary Figure 2. Trends in retail value of cigarettes and cigars/cigarillos in upper middle-income countries and smokeless tobacco in lower middle/low-income countries from 2007 to 2021

A **Retail value of cigarettes in China and the upper middle-income group excluding China**

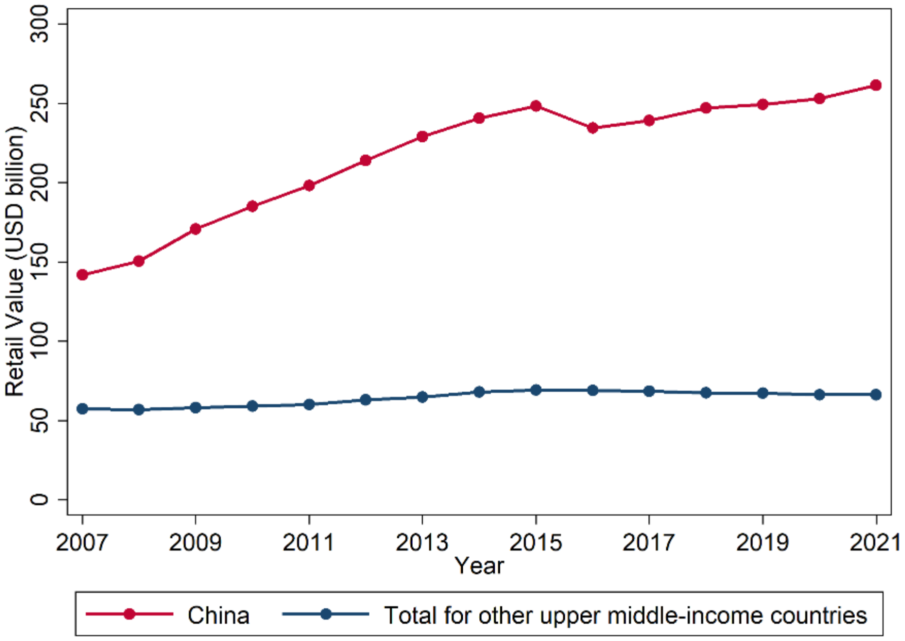

B **Retail value of cigars/cigarillos in China and the upper middle-income group excluding China**

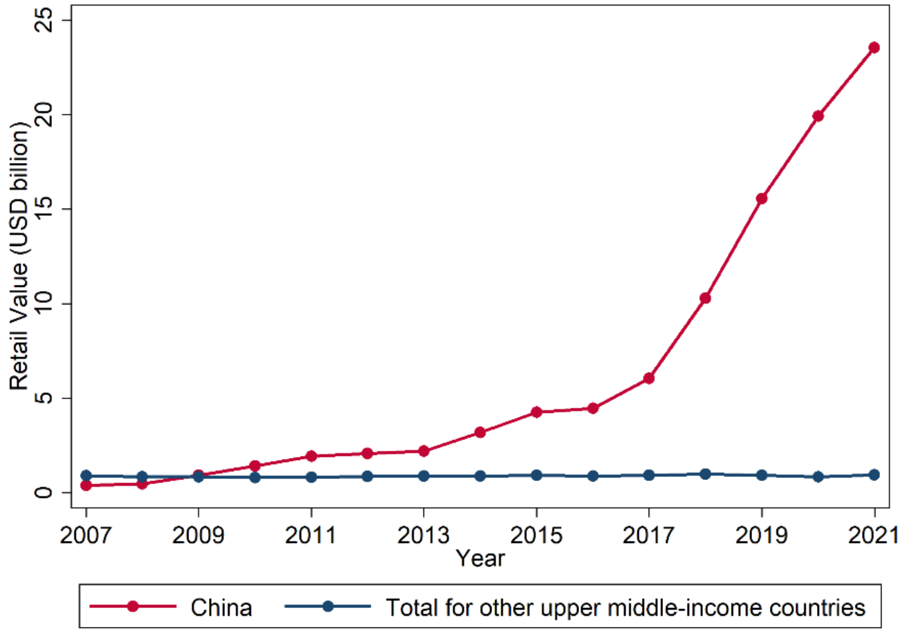

Supplementary Figure 2 continued on next page.

Supplementary Figure 2. (Continued)

C **Retail value of smokeless tobacco in India and the lower middle/low-income group excluding India**

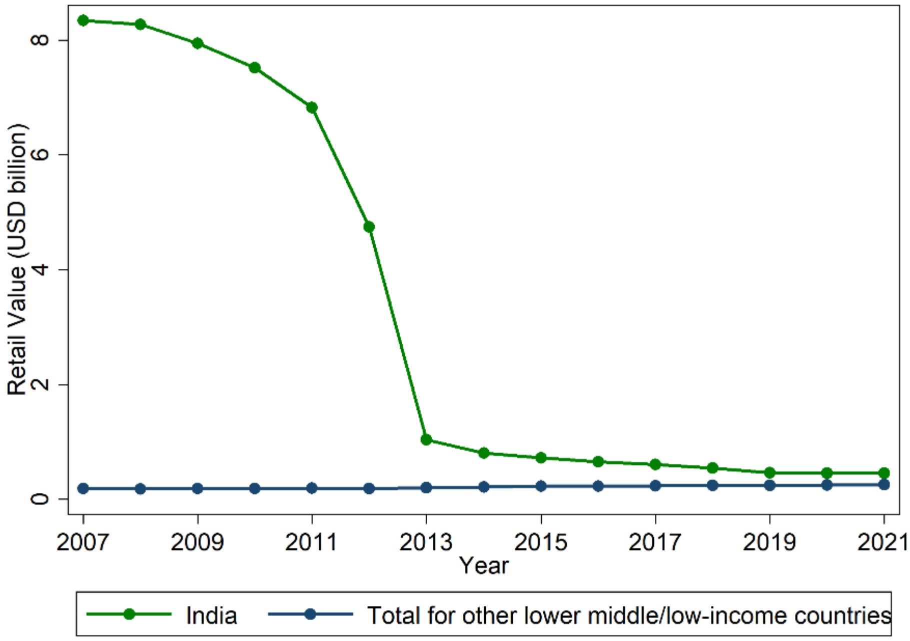

© 2024 Liu Y. and Filippidis F.T.
